# Functional outcomes and quality of life at 1-year follow-up after an open tibia fracture in Malawi: a multicentre, prospective cohort study

**DOI:** 10.1016/S2214-109X(23)00346-7

**Published:** 2023-09-01

**Authors:** Alexander Thomas Schade, Maureen Sabawo, Nohakhelha Nyamulani, Chikumbutso Clara Mpanga, Leonard Banza Ngoie, Andrew John Metcalfe, David G Lalloo, Jason J Madan, William James Harrison, Peter MacPherson

**Affiliations:** a Malawi–Liverpool-Wellcome Trust, Blantyre, Malawi; b Liverpool School of Tropical Medicine, Liverpool, UK; c Queen Elizabeth Central Hospital, Blantyre, Malawi; d Kamuzu University of Health Sciences, Blantyre, Malawi; e Kamuzu Central Hospital, Lilongwe, Malawi; f University of Warwick Medical School, Coventry, UK; g Countess of Chester NHS Foundation Trust, Chester, UK; h AO Alliance, Davos, Switzerland; i University of Glasgow, Glasgow, UK

## Abstract

**Background:**

Injuries are a major cause of disability globally and injury incidence is rapidly increasing, largely due to road traffic injuries in low-income and middle-income countries. Current estimates of the scale and consequences of disability from injury are largely based on modelling studies, with a scarcity of empirical evidence from severe injuries in low-income countries. We aimed to better understand the outcomes for individuals with open tibia fractures in Malawi.

**Methods:**

In this multicentre, prospective cohort study, adults (aged ≥18 years) with open tibia fractures were systematically recruited at six hospitals in Malawi (two tertiary hospitals and four district hospitals). Follow-up lasted at least 1 year, during which in-person follow-up reviews were done at 6 weeks, 3 months, 6 months, and 1 year post-injury. The primary outcome was function at 1 year post-injury, measured by the Short Musculoskeletal Functional Assessment (SMFA) score. Secondary outcomes included quality-adjusted life-years (QALYs; as determined via the European Quality of Life 5-Dimensions 3-Levels [EQ-5D-3L] survey) and fracture-related infection at 1 year post-injury. Multilevel regression models investigated associations between SMFA score, EQ-5D-3L, baseline factors, and orthopaedic management.

**Findings:**

Between Feb 12, 2021, and March 14, 2022, 287 participants were enrolled (median age 34 years [IQR 25–44]; 84% male). The most common mode of injury was road traffic injuries (194 [68%] of 287). Overall, 268 (93%) participants had debridement; of the 63 participants who were debrided in district hospitals, 47 (75%) had the procedure under local or no anaesthesia. Following substantial declines by 6 weeks after injury, function and quality of life had not recovered by 1 year post-injury for participants with Gustilo grade I–II fractures (posterior mean SMFA at 1 year: 10·5, 95% highest density interval [HDI]: 9·5–11·6; QALYs: 0·73, 95% HDI: 0·66–0·80) nor Gustilo grade III fractures (posterior mean SMFA at 1 year: 14·9, 95% HDI: 13·4–16·6; QALYs: 0·67, 95% HDI: 0·59–0·75). For all fracture grades, intramedullary nailing substantially improved function and quality of life at 1 year post-injury. Delayed definitive fixation after 5 days had 5-times greater odds of infection compared with early management within 2 days (adjusted odds ratio: 5·1, 95% CI 1·8–16·1; p=0·02).

**Interpretation:**

Adults with open tibia fractures in Malawi have poor function and quality of life in the 1 year following injury. Centralised orthopaedic surgical management, including early definitive fixation and intramedullary nailing for more severe injuries, might improve outcomes.

**Funding:**

Wellcome Trust.

**Translation:**

For the Chichewa translation of the abstract see Supplementary Materials section.

## Introduction

Injuries are the largest cause of death and disability for men aged 19–39 years, with 90% of injuries worldwide occurring in low-income and middle-income countries (LMICs) in 2018.^1^ Rates of injury and death are accelerating rapidly in LMICs due to economic growth, urbanisation, and increased road vehicle use.^2^ The evidence for the magnitude and consequences of disability from injury in LMICs is mostly based on modelling studies,^3^ with empirical evidence from low-income countries being scarce.

Open fractures are severe injuries where the bone pierces through the skin upon fracture. These fractures are a common cause of disability following road traffic injuries. The tibia is one of the most commonly injured long bones^4^ and, due to its superficial location, tibia fractures are highly susceptible to becoming open fractures.^5^ Open tibia fractures have devastating consequences for individuals and households in high-income countries,^6^ and, in LMICs, impacts are likely to be more severe. The little data available from LMICs show a 15% amputation rate, 18% infection rate, and 15% non-union rate related to these fractures, resulting in only 20% of patients being able to return to work at 1 year post-injury.^7^

Malawi is a low-income country situated in Africa with a population of 20 million, of whom 83% live in rural areas, and 50% live below the national poverty line.^8^ Malawi has one of the highest rates of road traffic deaths in the world,^3^ and orthopaedic trauma—including open tibia fractures—is rapidly increasing.^9^, ^10^ Open fracture care is mostly provided at secondary care level in district hospitals (which are typically rural, non-operative, and staffed by non-physician clinical officers with basic orthopaedic training^11^ as opposed to surgeons), and at tertiary care level in referral hospitals (which have access to investigations, operative management, and orthopaedic specialists). The majority (92%) of fracture care is delivered by orthopaedic clinical officers, and is non-operative.^5^ Open fractures should follow a standardised care pathway,^12^ which includes the early administration of antibiotics, surgical debridement—ie, the removal of all contaminated and devitalised tissue and washout of the open fracture in the operating theatre—and fracture immobilisation by internal fixation (such as intramedullary nail) or external fixation.

**Research in context**

**Evidence before this study**

We extended a previous systematic review to June 28, 2023. We searched PubMed for relevant papers published in English between database inception and June 28, 2023, using the search terms “outcomes” AND “open tibia fracture”. This search yielded 27 studies, most of which were from middle-income settings, and the four from low-income countries either focused on disasters or war zones or were from single tertiary referral centres. The scarce evidence available from these settings suggested that open tibia fractures might be likely to lead to severe impairment, but impacts are highly heterogeneous and potentially modifiable with improved orthopaedic management.

**Added value of this study**

This multicentre, prospective cohort study in Malawi in which adults with open tibia fracture were followed up for 1 year post-injury to assess functional and quality of life outcomes showed that open tibia fractures were common. Following substantial declines immediately after injury, Short Musculoskeletal Functional Assessment (SMFA) dysfunction scores had not recovered at 1-year follow-up for participants with Gustilo grade I–II fractures (posterior mean: 10·5, 95% highest density interval [HDI] 9·5 to 11·6) nor Gustilo grade III fractures (14·7, 13·2 to 16·2). Quality of life at 1 year post-injury was also substantially below baseline for participants with Gustilo grade I–II fractures (posterior mean quality-adjusted life-years: 0·73, 95% HDI 0·66 to 0·80) and Gustilo grade III fractures (0·67, 0·59 to 0·75). Fracture-related infection post-injury was common and was associated with delayed definitive operative management. Participants with Gustilo grade III injuries who received intramedullary nailing had a greater improvement (ie, less dysfunction) in functional outcomes at 1 year than did those who received external fixation (improvement in –11·2 SMFA dysfunction index [95% HDI –15·5 to –6·8]).

**Implications of all the available evidence**

Open tibia fractures are becoming increasingly common globally and are associated with significant disability. Global health strategies are needed to prevent and mitigate harms for individuals. Our findings show that district hospitals in Malawi are currently not providing optimal care for people with open fractures to maximise recovery. Operative trauma management in tertiary hospitals, including early definitive fixation and intramedullary nailing for more severe injuries, might improve outcomes of these severe injuries.

Patient-reported functional and quality of life outcomes are key to understanding and improving outcomes after open tibia fractures, as well as guiding health resource allocation. We hypothesised that, due to high-energy trauma, and scarce individual and health systems resources,^13^ adults with open tibia fractures in Malawi would have poor functional outcomes and quality of life in the 1 year after injury. We aimed to investigate the function and quality of life of participants with open tibia fractures at 1 year post-injury and to assess the impact of fracture severity and orthopaedic treatment modality on function, quality of life, and fracture-related infection.

## Methods

### Study design and participants

This prospective, multicentre cohort study was conducted in six hospitals in Malawi: two tertiary hospitals (Queen Elizabeth Central Hospital and Kamuzu Central Hospital), and four district hospitals (Dedza District Hospital, Ntcheu District Hospital, Balaka District Hospital, and Machinga District Hospital). Brief characteristics of each hospital are given in appendix 2 (pp 6–7). The study was approved by the College of Medicine Research and Ethics Committee in Malawi and the Liverpool School of Tropical Medicine in the UK. Written informed consent was obtained from all patients in the study. If potential participants were illiterate, consent forms were explained to them by study investigators and participants provided their fingerprint as an indication of informed consent in the presence of an independent witness, who also provided a signature. The study protocol has been published previously.^14^

Potential participants were systematically screened for study inclusion by health workers (orthopaedic clinical officers or orthopaedic surgeons in the six hospitals) who had received study-specific training at workshops. Eligible participants were adult patients (aged ≥18 years) who presented to hospital emergency departments with an open tibia fracture (as per The Arbeitsgemeinschaft für Osteosynthesefrage Foundation/Orthopaedic Trauma Association class 42)^15^ between Feb 12, 2021, and March 14, 2022. We excluded people who were unable to consent to study participation or were unable to complete patient-reported outcome questionnaires.

### Procedures

Open tibia fractures were confirmed by radiographs. Participants completed a detailed clinical and socio-demographic questionnaire. Questionnaire interviews were performed by study research assistants who were not affiliated with the six hospitals. Fracture Gustilo classification^16^ was documented by the most senior surgeon at each site. The study team did not intervene in clinical management or treatment decisions.

### Follow-up

In-person follow-up reviews were performed by the study research assistant at 6 weeks, 3 months, 6 months, and 1 year post-injury, coinciding with planned attendance at outpatient fracture clinics where possible. Short Musculoskeletal Functional Assessment (SMFA) and European Quality of Life 5-Dimensions 3-Levels (EQ-5D-3L) questionnaires were completed at each follow-up appointment and the presence of fracture-related infection was checked for. If participants did not attend a follow-up review appointment, then a telephone interview was undertaken to complete the SMFA and EQ-5D-3L questionnaires. If participants were not contactable by telephone, research assistants undertook home tracing and performed at-home interviews. Participants who could not be traced were considered to have been lost to follow-up.

### Outcomes

The primary outcome was SMFA dysfunction score at 1 year post-injury (which ranges from 0 [no functional impairment] to 100 [severe functional impairment])^17^ as determined via the SMFA questionnaire, which had been translated into and validated in Chichewa.^18^ Change in SMFA dysfunction was compared between baseline and 1 year post-injury. Secondary outcomes, assessed at 1 year post-injury, were the EQ-5D-3L index score (which ranges from –0·145 [quality of life worse than death] to 1 [perfect quality of life])^19^ and quality-adjusted life-years (QALYs) as determined via the EQ-5D-3L survey, which had been translated into and validated in Chichewa,^20^ and incidence of fracture-related infection.^21^

Baseline assessments were done by study research assistants in the hospital once participants were determined to be stable by the treating clinical team by asking participants to self-report their function and quality of life before injury using the SMFA and EQ-5D-3L questionnaires. The index utility tariffs for EQ-5D-3L scores were generated using the value set for the Zimbabwean population, as no set exists for Malawi.^22^ Fracture-related infection^21^ was confirmed by study investigators via inspection of the wound site and by reviewing medical notes for confirmatory clinical signs (eg, purulent discharge, presence of sinus or fistula, or wound breakdown) at each follow-up assessment.

### Statistical analysis

The target sample size was 125 participants, which would provide 80% power to detect a difference of 20% variance in SMFA index score between baseline and 1 year post-injury with an alpha of 0·01, allowing for 20% loss to follow-up. In the event that this target sample size was exceeded, we planned to recruit until the end of the study period (ie, 1 year).

The study is reported in accordance with STROBE guidelines, and the STROBE checklist is available in appendix 2 (pp 1–2). We summarised participants’ clinical and sociodemographic characteristics and compared these between those participants whose fractures were initially managed at the two tertiary hospitals and in the four district hospitals using Kruskal-Wallis and χ^2^ tests. To investigate trajectories in functional outcome and quality of life (via SMFA and EQ-5D-3L, respectively) following injury, we constructed Bayesian multilevel regression models, with inference drawn using Markov chain Monte Carlo sampling (appendix 2 pp 6–7).

As missing data were minimal, we performed a complete case analysis. To account for the fact that orthopaedic and surgical interventions are guided by injury severity, we fitted models separately for participants with Gustilo grade I or II fractures and participants with Gustilo grade III fractures. In this stratified analysis comparing function and quality of life outcomes by orthopaedic treatment modality, definitive fixation with amputation, external fixation, and plates for Gustilo I–II were excluded and plates for Gustilo III were excluded. These exclusions were made on the basis of the listed procedures being non-standard procedures or rarely performed for these grades of fracture. Models were fit using the R brms package as an interface to CmdStanR in R (version 4.3.1).^23^ We rescaled SMFA scores to range between 0 and 1, and modelled outcome variables using zero-one-inflated beta distributions. We included participant-level random intercepts, and adjusted for age and days to first surgical intervention a priori. 2000 post warmup posterior samples were drawn and summarised by their mean and 95% highest density interval. We calculated the cumulative SMFA points lost and QALYs in the 1 year post-injury by applying the trapezoid rule to integrate areas under curves of trajectories of posterior distributions. We compared the effects of orthopaedic treatments for Gustilo I–II and III injuries on SMFA and QALYs at 1 year post-injury and within this 1-year period, and estimated marginal effects for age and days to surgical intervention. We also compared function and quality of life at 1 year post-injury with baseline distributions. Logistic regression models were constructed to compare the effect of different orthopaedic treatments on the odds of fracture-related infection.

### Role of the funding source

The funder of the study had no role in study design, data collection, data analysis, data interpretation, or writing of the report.

## Results

**Figure 1:**
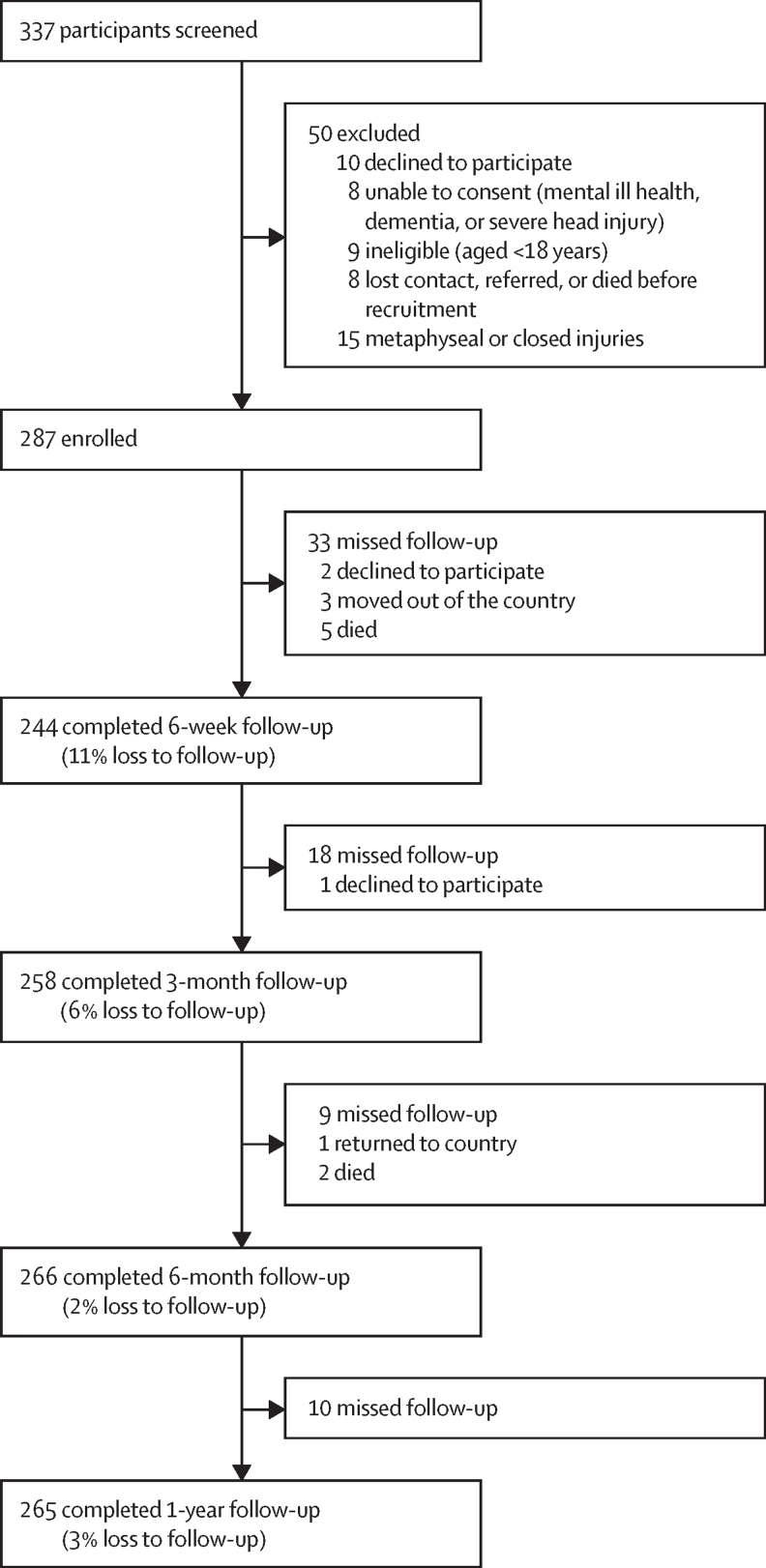
Study profile Participants lost to follow-up are shown at each stage (non-cumulative). The greater loss to follow-up at 6 weeks was due to the fact that, early in the study, some participants were not successfully traced. Once identified, the necessary measures were implemented to trace the participants, obtain their updated contact details, and ensure proper follow-up for the remaining study period, meaning that they attended later assessments.

Between Feb 12, 2021, and March 14 2022, 337 participants were screened, 313 were eligible and 287 were recruited across the six study sites. Overall, 224 (78%) participants were recruited from the two tertiary hospitals and 63 (22%) participants from the four district hospitals. At 1 year, there were a total of seven deaths, three withdrawals, and two participants who had moved to other countries (figure 1). The median age was 34 years (IQR 26–44), and 248 (86%) were male (table 1). Road traffic injuries were the most common mechanism of injuries and most commonly involved motorcycles or pedestrians. For the 224 participants treated at the tertiary hospitals, 91 (41%) presented directly to the tertiary hospital, and 64 (29%) were referred from a district hospital, 39 (17%) from a health-care centre, 24 (11%) from private institutions, four (2%) from faith-based institutions and two (1%) from other medical centres. Seven (58%) of the 12 participants with Gustilo III fractures who presented to district hospitals were referred to a tertiary hospital.

**Table 1:** Baseline participant and orthopaedic treatment characteristics

			**Tertiary hospitals (n=224)**	**District hospitals (n=63)**	**Total (n=287)**	**p value**
**Baseline data**						
Median age (IQR), years			34 (26–44)	36 (25–49)	34 (26–45)	0·55
Sex						
	Male		198 (88%)	50 (79%)	248 (86%)	0·10
	Female		26 (12%)	13 (21%)	39 (14%)	
Current smoker			44 (20%)	10 (16%)	54 (19%)	0·62
Comorbidities			12 (5%)	10 (16%)	22 (8%)	0·01
Other injuries			78 (35%)	9 (14%)	87 (30%)	<0·01
Mechanism of fracture			..	..	..	<0·01
	Road traffic injury		154 (68%)	44 (70%)	197 (69%)	..
		Motorcycle	64 (28%)	17 (27%)	81 (28%)	..
		Pedestrian	53 (23%)	22 (35%)	74 (26%)	..
		Bicycle	14 (5%)	4 (6%)	18 (6%)	..
		Car	20 (9%)	0	20 (7%)	..
		Minibus or heavy goods vehicle	3 (1%)	1 (2%)	3 (1%)	..
	Assault		41 (18%)	4 (6%)	44 (15%)	..
	Fall, ≤2 m		6 (3%)	1 (2%)	7 (2%)	..
	Fall, >2 m		3 (1%)	1 (2%)	4 (1%)	..
	Blunt force		17 (8%)	7 (11%)	24 (8%)	..
	Sport		3 (1%)	4 (6%)	7 (2%)	..
	Gunshot		2 (1%)	0	2 (1%)	..
	Work-related		0	1 (2%)	1 (<1%)	..
	Animal bite		0	1 (2%)	1 (<1%)	..
Gustilo classification			..	..	..	<0·01
	Grade I		45 (20%)	31 (51%)	76 (26%)	..
	Grade II		69 (31%)	18 (30%)	87 (30%)	..
	Grade III (type A, B or C)		107 (47%)	12 (20%)	119 (41%)	..
	Missing data		5 (2%)	0	5 (2%)	..
**Initial orthopaedic management**						
Median days from injury to debridement (IQR)			1 (1–3)	1 (0–1)	1 (0–3)	<0·01
Debridement performed			208 (93%)	60 (95%)	268 (93%)	
Anaesthetic used for debridement			..	..	..	<0·01
	No anaesthesia		2 (1%)	6 (10%)	3 (1%)	..
	Local anaesthesia		5 (2%)	39 (65%)	44 (17%)	..
	Spinal anaesthesia		168 (81%)	8 (13%)	176 (67%)	..
	General anaesthesia		33 (16%)	7 (12%)	40 (15%)	..
Documented surgeon for debridement			..	..	..	0·33
	Non-medical personnel		1 (<1%)	6 (7%)	4 (2%)	..
	Orthopaedic clinical officer or trainee clinical officer		45 (22%)	57 (93%)	102 (39%)	..
	Non-orthopaedic doctor		12 (6%)	0	12 (5%)	..
	Orthopaedic surgeon		144 (72%)	0	144 (55%)	..
	Missing data		6 (3%)	0	6 (2%)	..
Definitive orthopaedic management*						
Median days from injury to definitive fixation (IQR)			4 (1–9)	NA	4 (1–9)	NA
Primary orthopaedic fixation			..	..	..	<0·01
	Plaster of Paris		28 (12%)	55 (95%)	82 (29%)	..
	Intramedullary nail†		112 (49%)	0	112 (39%)	..
	External fixator		69 (30%)	1 (2%)	70 (24%)	..
	Plate		4 (2%)	0	4 (2%)	..
	Amputation		16 (7%)	2 (3%)	18 (6%)	..
Days from injury to wound cover			2 (1–8)	NA	2 (1–8)	NA
Type of wound cover			..	..	..	0·06
	Primary closure		184 (80%)	56 (97%)	240 (84%)	..
	Flap or split skin graft		19 (8%)	0	19 (7%)	..
	Secondary healing		10 (4%)	0	10 (4%)	..
	Amputation		16 (7%)	2 (3%)	18 (6%)	..

Comorbidities were defined as: diabetes, heart disease, lung disease, neurological disease, hypertension, or previous mobility issues. Other injuries were defined as: head, spinal, thorax, abdomen, pelvis, upper limb, or other lower limb injuries. Orthopaedic surgeon was defined as an orthopaedic registrar, resident, or consultant. For initial orthopaedic management, seven (3%) people in tertiary hospitals who reported having debridement under local or no anaesthesia had this performed in district hospitals. For definitive orthopaedic management, the participant numbers differ from the column totals, and instead are n=229 (80%) for tertiary hospitals and n=58 (20%) for district hospitals to a total of n=287. NA=not applicable.

* Five patients were transferred from district hospitals to tertiary hospitals for definitive management (fixation and wound cover).

† Intramedullary nail refers to the Surgical Implant Generation Network (known as SIGN) intramedullary nail.

283 (99%) of 287 participants received antibiotics, with 255 receiving ceftriaxone and 33 receiving tetanus prophylaxis. Median time from injury to initial debridement was 1 day (IQR 0–3). In district hospitals, 47 (75%) of 60 initial debridements were done under local anaesthesia or no anaesthesia; whereas, in tertiary hospitals, 201 (100%) were done under general or spinal anaesthesia. In tertiary hospitals, 144 (72%) debridements were documented to have an orthopaedic surgeon involved; however, in district hospitals, 57 (93%) had an orthopaedic clinical officer documented as being involved. Overall, 112 (40%) participants underwent intramedullary nailing, 70 (24%) received external fixation, four (2%) received plating, 82 (29%) had plaster of Paris (POP), and 18 (6%) required amputation. 240 participants had primary closure of their wounds. Median time to initial surgical fixation was 5 days (IQR 1–10) for Gustilo grade I–II fractures and 3 days (IQR 1–8) for Gustilo grade III fractures.

**Figure 2:**
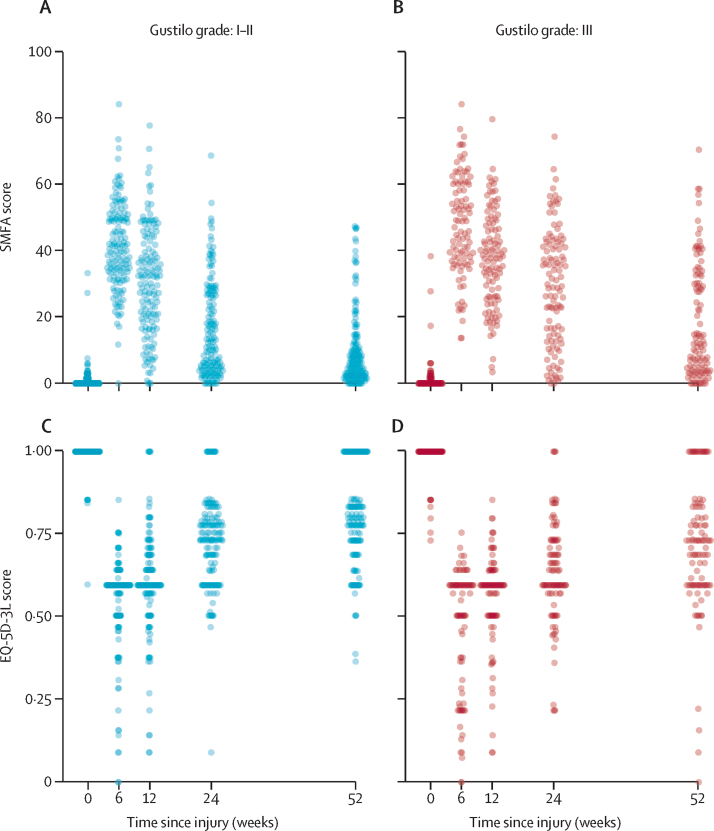
Empirical SMFA and EQ-5D-3L scores at 1 year post-injury in participants with open tibia fracture Scatterplot of SMFA-dysfunction index scores and EQ-5D-3L scores for participants with Gustilo grade I and II injuries (A and C) and Gustilo grade III injuries (B and D). EQ-5D-3L=European Quality of Life 5-Dimensions 3-Levels. SMFA=Short Musculoskeletal Functional Assessment.

At baseline, nearly all participants reported excellent pre-injury functional scores (median SMFA: 0, IQR 0–0·75) and quality of life (median EQ-5D-3L: 1, IQR 1–1; figure 2). However, there were substantial reductions in both measures by week 6 post-injury. By 1-year follow-up, overall median SMFA was 7·35 (IQR 2·9·1–19·1) and median EQ-5D-3L was 0·78 (IQR 0·66–1·00). Over the subsequent follow-up period, participants in both Gustilo grade groups (I–II and III) had slow recovery of both SMFA and EQ-5D-3L scores; however, by 1 year post-injury, function and quality of life scores were markedly worse among participants with Gustilo grade III fractures. At 1 year, 66 (25%) of 260 participants had an EQ-5D-3L score of 1, and 24 (9%) had an SMFA score of 0.

After exclusion of non-standard or rare treatment modalities (missing Gustilo classification [n=5], amputation cases [n=5], external fixation [n=17], cases involving plates [n=2] for grade I fractures, and cases involving plates [n=3] for grade III fractures), 251 participants were included in the models. Posterior distributions showed that, at 1 year post-injury, SMFA dysfunction scores and quality of life scores remained substantially below baseline for participants with Gustilo grade I–II fractures and with Gustilo grade III fractures (table 2).

**Table 2:** Model-estimated SMFA and EQ-5D-3L scores following open tibia fracture, and comparison between orthopaedic treatments

	**SMFA (posterior mean, 95% highest density interval)**					**EQ-5D-3L (posterior mean, 95% highest density interval)**				
	Week 0	Week 6	Week 12	Week 24	Week 52	Week 0	Week 6	Week 12	Week 24	Week 52
**Overall**										
Gustilo grade I–II	1·0 (0·7 to 1·3)	39·4 (37·4 to 41·4)	29·0 (27·2 to 30·8)	17·7 (16·3 to 19·2)	10·5 (9·5 to 11·6)	0·98 (0·96 to 0·99)	0·56 (0·54 to 0·57)	0·61 (0·59 to 0·63)	0·71 (0·69 to 0·73)	0·80 (0·78 to 0·82)
Gustilo grade III	1·6 (1·1 to 2·1)	48·7 (46·1 to 51·4)	37·4 (35·1 to 39·7)	24·1 (22·1 to 26·1)	14·9 (13·4 to 16·6)	0·97 (0·96 to 0·98)	0·49 (0·47 to 0·51)	0·55 (0·53 to 0·57)	0·65 (0·63 to 0·67)	0·73 (0·70 to 0·75)
Difference (grade III *vs* grade I–II)	0·6 (0·2 to 1·1)	9·3 (5·8 to 12·7)	8·4 (5·3 to 11·6)	6·4 (4·0 to 8·8)	4·4 (2·7 to 6·1)	−0·01 (−0·01 to 0·00)	−0·06 (−0·09 to −0·04)	−0·06 (−0·09 to −0·04)	−0·06 (−0·09 to −0·04)	−0·07 (−0·10 to −0·05)
**Gustilo grade I–II injury**										
Nail	0·5 (0·2 to 1·3)	37·0 (33·3 to 40·6)	22·0 (19·3 to 24·8)	11·2 (9·3 to 13·4)	7·3 (5·8 to 8·9)	0·98 (0·96 to 0·99)	0·58 (0·55 to 0·60)	0·63 (0·60 to 0·65)	0·74 (0·72 to 0·77)	0·82 (0·79 to 0·84)
POP (tertiary)	1·5 (0·7 to 2·6)	39·0 (33·4 to 44·8)	25·6 (19·8 to 31·4)	11·5 (8·5 to 15·1)	7·2 (4·7 to 10·1)	0·98 (0·96 to 0·99)	0·58 (0·54 to 0·62)	0·64 (0·60 to 0·68)	0·77 (0·73 to 0·81)	0·87 (0·82 to 0·91)
POP (district)	2·5 (1·5 to 4·1)	47·5 (43·1 to 51·7)	37·5 (33·5 to 41·6)	20·9 (17·7 to 24·3)	11·9 (9·6 to 14·4)	0·98 (0·96 to 0·99)	0·54 (0·51 to 0·57)	0·59 (0·56 to 0·62)	0·72 (0·69 to 0·75)	0·81 (0·77 to 0·84)
Difference (dPOP *vs* tPOP)	1·1 (−0·4 to 2·8)	8·7 (1·0 to 16·1)	11·8 (4·4 to 19·2)	9·4 (4·7 to 14·1)	4·8 (1·0 to 8·2)	0·00 (−0·01 to 0·00)	−0·04 (−0·09 to 0·00)	−0·05 (−0·09 to 0·00)	−0·05 (−0·09 to −0·01)	−0·06 (−0·10 to −0·01)
Difference (nail *vs* tPOP)	−1·0 (−2·2 to 0·1)	−2·0 (−9·2 to 5·1)	−3·8 (−10·4 to 2·7)	−0·4 (−4·5 to 3·6)	0·1 (−3·1 to 3·2)	0·00 (−0·01 to 0·00)	0·00 (−0·05 to 0·04)	−0·01 (−0·06 to 0·03)	−0·03 (−0·06 to 0·01)	−0·05 (−0·09 to −0·01)
Difference (nail *vs* dPOP)	−2·0 (−3·7 to −0·8)	−10·6 (−16·8 to −4·5)	−15·5 (−21·1 to −10·0)	−9·7 (−13·9 to −5·7)	−4·6 (−7·6 to −1·6)	0·00 (0·00 to 0·00)	0·04 (0·00 to 0·07)	0·04 (0·00 to 0·07)	0·03 (−0·01 to 0·06)	0·01 (−0·03 to 0·05)
**Gustilo grade III injury**										
External fixator	1·4 (0·8 to 2·3)	52·2 (48·0 to 56·4)	41·2 (37·1 to 45·2)	35·0 (30·8 to 39·1)	19·7 (16·3 to 23·3)	0·96 (0·92 to 0·98)	0·46 (0·42 to 0·50)	0·52 (0·49 to 0·56)	0·59 (0·56 to 0·63)	0·69 (0·65 to 0·73)
Nail	0·6 (0·2 to 1·6)	39·0 (34·6 to 43·5)	28·2 (24·6 to 32·1)	15·5 (12·9 to 18·5)	8·4 (6·5 to 10·6)	0·97 (0·93 to 0·99)	0·55 (0·52 to 0·59)	0·61 (0·58 to 0·65)	0·68 (0·65 to 0·71)	0·77 (0·73 to 0·81)
Amputation	2·7 (1·0 to 5·6)	48·3 (38·9 to 57·8)	41·6 (33·3 to 50·4)	38·5 (30·9 to 47·2)	31·9 (25·0 to 39·9)	0·95 (0·91 to 0·98)	0·45 (0·40 to 0·51)	0·52 (0·46 to 0·58)	0·59 (0·53 to 0·65)	0·68 (0·61 to 0·75)
POP (district)	4·7 (1·3 to 10·9)	46·8 (31·6 to 61·7)	42·8 (28·3 to 58·2)	37·1 (23·6 to 52·2)	28·8 (17·5 to 42·6)	0·95 (0·90 to 0·98)	0·52 (0·42 to 0·62)	0·59 (0·49 to 0·68)	0·65 (0·56 to 0·74)	0·72 (0·63 to 0·82)
Difference (dPOP *vs* amputation)	1·9 (−2·3 to 8·1)	−2·0 (−19·0 to 16·2)	1·0 (−16·3 to 18·0)	−1·5 (−17·7 to 15·8)	−3·1 (−17·4 to 12·4)	0·00 (−0·05 to 0·03)	0·07 (−0·04 to 0·17)	0·07 (−0·04 to 0·17)	0·06 (−0·04 to 0·16)	0·05 (−0·07 to 0·16)
Difference (external fixator *vs* amputation)	−1·3 (−4·3 to 0·5)	3·9 (−6·7 to 14·6)	−0·5 (−10·6 to 9·5)	−3·5 (−13·4 to 5·6)	−12·3 (−21·2 to −4·3)	0·00 (−0·01 to 0·03)	0·01 (−0·06 to 0·07)	0·00 (−0·06 to 0·07)	0·01 (−0·06 to 0·07)	0·01 (−0·07 to 0·09)
Difference (nail *vs* amputation)	−2·0 (−5·0 to −0·2)	−9·3 (−19·9 to 1·7)	−13·4 (−23·4 to −3·8)	−23·0 (−32·3 to −14·5)	−23·4 (−31·7 to −16·2)	0·01 (0·00 to 0·04)	0·10 (0·04 to 0·16)	0·10 (0·03 to 0·16)	0·09 (0·03 to 0·15)	0·09 (0·01 to 0·16)
Difference (external fixator *vs* dPOP)	−3·2 (−9·4 to 0·2)	5·8 (−10·2 to 21·6)	−1·5 (−17·7 to 14·1)	−2·2 (−17·6 to 12·1)	−9·1 (−23·5 to 3·1)	0·00 (−0·01 to 0·06)	−0·06 (−0·16 to 0·04)	−0·07 (−0·16 to 0·04)	−0·06 (−0·15 to 0·04)	−0·03 (−0·14 to 0·07)
Difference (nail *vs* dPOP)	−3·9 (−10·2 to −0·5)	−7·6 (−22·7 to 7·8)	−14·5 (−30·5 to 0·5)	−21·6 (−37·1 to −7·4)	−20·4 (−34·5 to −8·8)	0·01 (−0·01 to 0·06)	0·03 (−0·07 to 0·13)	0·03 (−0·07 to 0·13)	0·03 (−0·06 to 0·13)	0·05 (−0·06 to 0·14)
Difference (nail *vs* external fixator)	−0·7 (−1·7 to 0·4)	−13·3 (−20·2 to −6·3)	−13·0 (−19·2 to −6·6)	−19·6 (−24·9 to −13·9)	−11·2 (−15·5 to −6·8)	0·01 (0·00 to 0·02)	0·09 (0·05 to 0·14)	0·09 (0·05 to 0·13)	0·09 (0·05 to 0·13)	0·08 (0·03 to 0·13)

Nail refers to the Surgical Implant Generation Network (known as SIGN) intramedullary nail. For all models, age is held constant at its mean, and days to initial surgical intervention at its median, and included participant-level random effects. EQ-5D-3L=European Quality of Life 5-Dimensions 3-Levels. POP=plaster of Paris. dPOP=plaster of Paris applied in district hospitals. tPOP=plaster of Paris applied in tertiary hospitals. SMFA=Short-Musculoskeletal Assessment Score.

**Figure 3:**
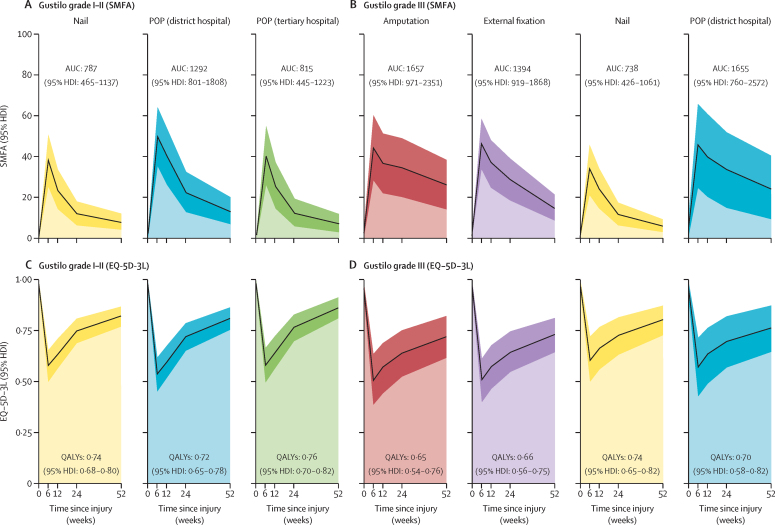
Modelled cumulative impact of open tibia fracture and orthopaedic management on function and quality of life Posterior predictions from Bayesian multilevel regression model, with age held constant at its mean, and days to initial surgical intervention at its median, plus participant-level random effects. AUC=area under the curve. EQ-5D-3L=European Quality of Life 5-Dimensions 3-Levels. HDI=highest density interval. POP=plaster of Paris. QALY=quality-adjusted life-year. SMFA=Short Musculoskeletal Functional Assessment.

Comparisons of orthopaedic treatments showed that participants with Gustilo grade I–II injuries who received an intramedullary nail had similar SMFA dysfunction scores at 1 year post-injury to participants who were managed with POP in a tertiary hospital, but had improved scores when compared with participants who were managed with POP in a district hospital (table 2). The mean posterior difference at 1 year in SMFA score comparing participants treated with intramedullary nail versus POP in district hospitals was –4·6 SMFA points (95% highest density interval –7·4 to –1·6). By contrast, there was no difference in EQ-5D-3L scores when comparing participants with Gustilo grade I–II fractures with intramedullary nail versus POP in district hospitals. Despite this finding, there was evidence that participants treated with intramedullary nail reported higher quality of life than those treated with POP in tertiary hospitals. Over the 1 year post-injury, participants with Gustilo grade I–II fractures who were treated with POP in district hospitals had a larger cumulative loss in function (SMFA area under the curve) then did those treated with POP at tertiary hospitals or intramedullary nail (figure 3).

Participants with Gustilo grade III fractures had substantially worse function and quality of life at 1 year post-injury than did participants with grade I–II fractures. However, there was strong evidence that outcomes differed by orthopaedic treatment modality. At 1 year, participants who received intramedullary nailing fared considerably better in terms of SMFA and EQ-5D-3L scores than did participants whose limb was amputated or underwent external fixation (table 2). Cumulatively, of participants with a Gustilo grade III fracture, those treated with intramedullary nails had the least loss of function (figure 3).

79 (28%) of 287 participants developed fracture-related infection, with seven (9%) of 76 Gustilo grade I injuries, 15 (17%) of 87 grade II injuries, and 54 (45%) of 119 grade III injuries becoming infected. Five participants were missing Gustilo classification (three patients with fracture-related infection but Gustilo classification missing). As infection mostly occurred in grade III injuries, we restricted regression analysis to this group. Median empirical SMFA scores for grade III injuries at 1 year post-injury were 27·9 (IQR 7·2–38·7) for those with fracture-related infection and 6·9 (IQR 2·9–14·5) for those without fracture-related infection. Similarly, median empirical EQ-5D-3L scores at 1 year for grade III injuries were 0·65 (IQR 0·59–0·73) versus 0·78 (IQR 0·69–0·86) for those with and without fracture-related infection, respectively. Participants who underwent external fixation were more likely to develop fracture-related infection than were those who underwent intramedullary treatment (table 3). Delayed definitive fixation after 5 days had 5-times greater odds of infection than did early management within 2 days (adjusted odds ratio: 5·1 [95% CI 1·8–16·1]; p=0·02).

**Table 3:** Associations with fracture-related infection for Gustilo grade III injuries

	**Fracture-related infection, n (%)**	Adjusted odds ratio for infection*	**95% CI**	**p value**
**Time to debridement**				
≤12 h	12/27 (44%)	Ref	Ref	0·32
>12 to ≤24 h	8/21 (38%)	0·7	0·2–2·4	..
1–3 days	14/28 (50%)	1·2	0·4–3·6	..
3–7 days	9/15 (60%)	1·8	0·5–7·1	..
>7 days	8/11 (73%)	3·6	0·8–20·2	..
**Type of fixation**				
Intramedullary nail	11/43 (26%)	Ref	Ref	0·02
External fixation	29/51 (57%)	4·1	1·7–10·4	..
**Time to definitive fixation**				
≤2 days	7/29 (24%)	Ref	Ref	0·05
>2 to ≤5 days	18/35 (50%)	3·4	1·2–10·6	..
>5 days	22/30 (53%)	5·1	1·8–16·1	..
**Type of wound cover**				
Primary wound closure	22/75 (34%)	Ref	Ref	<0·01
Soft tissue reconstruction	11/19 (68%)	5·1	1·7–16·8	..
Secondary wound healing	8/10 (80%)	10·1	2·3–72·9	..
**Timing to wound cover**				
≤2 days	8/26 (31%)	Ref	Ref	0·03
>2 to ≤5 days	13/29 (45%)	2·6	0·9–8·1	..
>5 days	20/35 (57%)	3·9	1·4–11·5	..

Intramedullary nail refers to the Surgical Implant Generation Network (known as SIGN) intramedullary nail.

* Adjusted for age, smoking status, and other injuries. For type and timing of fixation: plate (n=2), plaster of Paris (n=7), and amputation (n=15) were removed. For type and timing of wound cover: amputation (n=15) was removed.

## Discussion

In this multicentre, prospective cohort study in Malawi, adults with an open tibia fracture had very poor musculoskeletal function and quality of life at 1 year post-injury. Our findings show that outcomes could be improved by centralising care to tertiary hospitals, reducing time to definitive fixation, and by use of intramedullary nails for more severe injuries. Injuries cause more than 220 million disability-adjusted life-years to be lost each year in LMICs, which is higher than that for ischaemic heart disease; cancer; or tuberculosis, HIV, and malaria combined.^4^ This disability is largely preventable through injury prevention schemes, but could also be improved through trauma care systems that are accessible and of good quality.^24^ Open fractures are a common and severe form of injury worldwide and this study provides evidence that, in resource-constrained settings, complex injuries should primarily be managed in tertiary hospitals.

Evidence from a qualitative study exploring disability following an open tibia fracture in Malawi suggests that participants still suffer with pain and immobility 10 years after injury.^25^ Despite this finding, in our analysis, participants with Gustilo III open tibia fractures in Malawi had better EQ-5D-3L scores at 1 year than did people with similar injuries in the UK.^7^ This outcome could be due to key differences between the two studies. The UK study recruited older participants, used a different EQ-5D-3L tariff, and only included severe open fractures with open wounds. In the present study, SMFA functional index scores for open tibia fracture in Malawi were worse at 6 weeks post-injury and similar at 1 year compared with patients with femoral shaft fractures in Malawi.^26^ Since our findings show that early operative management of open tibia fractures by specialists in tertiary centres improves function and impairment, it is important that participants with these severe injuries should be quickly referred to central facilities to minimise poor function post-injury. Further qualitative studies among orthopaedic staff in district hospitals are needed to understand if the lack of referral of such severe injuries was due to patient-level factors, or environmental, or health-care decision-making factors (ie, clinical officers not referring to tertiary hospitals).

Capacity to administer operative orthopaedic care in Malawi is restricted to the tertiary hospitals, with 99·7% of operative fixation for open tibia fractures occurring in these facilities. In the present study, participants who received intramedullary nailing had substantially better functional outcomes and quality of life at 1 year post-surgery than did participants treated non-operatively with POP for Gustilo I–II injuries. For participants with Gustilo III injuries, those who received intramedullary nailing also had better functional outcomes than their counterparts treated with external fixation. A randomised controlled trial among adults with Gustilo type IIIA or type IIIB open tibia fractures in 20 trauma centres in the USA showed that external ring fixation had a higher complication rate than did internal fixation (62% *vs* 44%, respectively).^27^ If open tibia fractures were prioritised over other orthopaedic injuries in tertiary hospitals, this decision might cause delayed and non-operative management for other orthopaedic injuries. A detailed cost and implementation feasibility analysis of intramedullary nailing is required to determine whether it is feasible to offer intramedullary nailing for all open tibia fractures in Malawi and in other LMICs.

The incidence of fracture-related infection in the present study was higher than historical data on open tibia fractures from high-income countries^16^ and from another study (recruited 2015–17) on adults with open tibia fractures in Tanzania.^28^ Specifically, infection was more common and was associated with very poor function and quality of life in participants with more severe fracture grades, with 45% of grade III injuries showing fracture-related infection (*vs* 9% for grade I and 17% for grade II). Type and timing of fixation were important determinants of infection, with people who received intramedullary nailing within 24 h having the lowest risk of infection. Further microbiology studies are required to determine if the pathogens are similar to those found in open fractures in high-income countries and the role of potential antibiotic resistance in Malawi.

The *Lancet* Commission on Global Surgery advocates for debridement to be available at any district hospital due to the importance of early debridement in improving outcomes.^29^ In this study, in district hospitals, 75% of people with open tibia fractures had debridement done under local or no anaesthetic, and 95% were treated with POP by orthopaedic clinical officers. The impact of rapid surgical debridement on infection risk remains controversial, with a meta-analysis of Gustilo grade III open fractures suggesting a progressive increase in the risk of infection with time after 12 h or 24 h post-injury, compared with before these 12 h or 24 h timepoints. Other cohort studies from high-income countries^30^ and our results suggest that, in this setting, fracture-related infection was only marginally reduced by debridement within 48 h compared with after 48 h. This finding is possibly due to the heterogeneity of the debridement procedure in terms of anaesthesia and surgical expertise. In high-income countries, high-quality debridement—ie, adequate anaesthesia and specialist expertise—might result in improved outcomes.^31^ Further evidence is needed to understand whether, for severe open tibia fracture, delayed debridement with earlier fixation in tertiary hospitals by trained orthopaedic surgeons would result in better function and lower infection rates for people in LMICs.

To our knowledge, this is the first large prospective study to investigate outcomes after open fractures in a low-income country, and we achieved a high level of follow-up under challenging conditions—including patient relocation, expensive follow-up transport, low coverage of mobile phone use, the COVID-19 pandemic, a national cholera outbreak, and severe cyclone and flooding events in Malawi due to the climate crisis. Previous studies from low-income countries focused on single centres with a low number of participants with open tibia fractures.^32^, ^33^ Several limitations of the present study should be noted. First, the present study is susceptible to selection bias, which could have underestimated the burden of disability after an open tibia fracture because of the recruitment of participants in district and tertiary hospitals exclusively. Further qualitative studies in the community could explore health-seeking behaviours after injury, but our informal studies suggest that almost all people with open fractures reach hospital. Furthermore, it is possible that patients with severe head injuries might have died before hospital admission^34^ and would not be able to complete patient-reported outcomes. Second, severe injuries not captured by the Gustilo classification were more likely to be managed with external fixation rather than intramedullary nailing or wound reconstruction rather than primary closure. The Gustilo classification is also subject to issues of misclassification, with suboptimal inter-observer agreement.^35^ Third, there is also potential for measurement biases, with the functional and quality of life scores not capturing all domains that are important to surgeons, and patients and their relatives. Further studies are need to develop tools that are appropriate and relevant to open fractures in this setting. Fourth, we were not able to investigate the effects of social support and physiotherapy services, which are scarce in LMICs.^36^ Given that rehabilitation programmes are known to impact function and quality of life in high-income countries, this component should be investigated in future studies in LMICs. Fifth, although participants of the present study were followed up for 1 year post-injury, the benefits of operative or non-operative management might accrue over a longer time period. Finally, despite exceeding recruitment targets, this study was not powered for the sub-analysis of treatments and infection and these results should be interpreted with caution. We have shown that it is feasible to conduct high-quality prospective follow-up of participants with complex injuries in a low-resource setting. Randomised trials comparing intramedullary nailing against external fixation are now required to provide stronger evidence for these interventions in low-income countries.

In summary, our findings show that most people with open tibia fractures in Malawi have poor function and quality of life at 1 year post-injury. We found that there was scarce surgical capacity in district hospitals for these injuries, and that participants had substantially improved outcomes if they received definitive early fixation with intramedullary nailing in tertiary hospitals. To improve outcomes from such injuries, we recommend upscaling orthopaedic treatment in Malawi hospitals with specialist surgical services, supported by effective patient transport, rehabilitation, and social support structures.

## Data sharing

The individual de-identified participant data and code to replicate analysis are available online.

## Declaration of interests

We declare no competing interests. AJM is an investigator on three National Institute for Health and Care Research-funded trials (Chief Investigator for START:REACTS and RACER-Knee and co-investigator for RACER-Hip), for which Stryker, a medical device company, fund some treatment costs and some imaging costs. For all these studies, the full independence of the study team is protected by legal agreements and they have no bearing on the presented study.

## Supplementary Materials

Chichewa translation of the abstract
