# Supplementary material for: Functional outcomes and quality of life at 1-year follow-up after an open tibia fracture in Malawi: a multicentre, prospective cohort study
Source: Lancet Glob Health. 2023 Sep 1;11(10):e1609–18. doi: 10.1016/S2214-109X(23)00346-7 (PMC10509037; doi:10.1016/S2214-109X(23)00346-7)
Supplement: Chichewa translation of the abstract [file mmc1.pdf]

THE LANCET
Global Health

Supplementary appendix 1

This translation in Chichewa was submitted by the authors and we reproduce it as supplied. It has not been peer reviewed. *The Lancet*'s editorial processes have only been applied to the original in English, which should serve as reference for this manuscript.

Kutanthauzila kwa mu Chichewa uku kwachokela kwa alembi a nkhaniyi ndipo tayipeleka kwa inu m'mene tinayilandilila. Mawu a mu Chichewa sanawunikilidwenso kapena kukonzedwa. Nkhani ya mu Chingelezi yokha ndi imene yadutsa mu ukonzi wa Lancet, kotelo kuti nkhani ya mu Chingeleziyi ndi imene ikuyimilila mokwanila nkhani yonse imene yalembedwa.

Supplement to: Schade AT, Sabawo M, Nyamulani N, et al. Functional outcomes and quality of life at 1-year follow-up after an open tibia fracture in Malawi: a multicentre, prospective cohort study. *Lancet Glob Health* 2023; published online Sept 1. https://doi.org/10.1016/S2214-109X(23)00346-7.

Mwachidule

Chiyambi

Kuvulala ndi njira imodzi yayikulu imene imayambisa ulumali padziko lonse lapansi ndipo zochitika zikuchulukirachulukira, makamaka chifukwa cha ngozi za pamsewu mu mayiko ovutikitsitsa. Umboni wa ulumali kamba ka kuvalala umachokera ku maphunziro achitsanzo omwe amachitika, koma palibe umboni wotsimikizira kuchokera pa kuvulala kwambili m'mayiko ovutikitsitsa. Chifukwa cha ichi, kuti timvetsetse bwino zotsatira za anthu omwe ali ndi vuto la kuthyoka ndi kutulukila kwa fupa la mwendo, tidachita kafukufufuku ku Malawi.

NDONDOMEKO

Anthu azaka 18 ndikuposela apo amene anabvulala (kuthyoka ndi kutulukila fupa la mwendo) analowa mu kafukufukuyu kutsatila ndondomeko mu zipatala zisanu ndi chimodzi mu dziko lino la Malawi (zipatala zIzikulukulu ziwili ndi zipatala za maboma zinanayi) omwe anatsatilidwa kwa chaka chimodzi, ndipo anayamba kusatilidwa pa masabata asanu ndi limodzi, miyezi itatu, miyezi isanu ndi limodzi komaso kupyola chaka chimodzi. Zotsatira zoyambirira zinali ntchito, zimene zinayezadwa potengela magwilidwe ntchito a minyewa/minofu ndi mafupa, zotsatila zachiwiri zimakhuza kusitha kwa moyo pakupita kwa nthawi komaso kuonongeka kwa chilonda. Kafukufukuyi masiyanitsa magwilidwe antchito ngozi isanachitike ndi pakadali pano.

ZOTSATIRA

Pakati pa mwezi wa February 12, 2021 ndi march 14, 2022, otenga mbali 287 analowa kafukufuku, anthuwa ndi azaka zapakati pa 34 (25-44). 84% azibambo. Pomwe njira imodzi inali ngozi zapanseu (194(68% mwa anthu 287) Onse, (n-268) 93% mwa otenga mbali anatsukidwa mabala, otenga mbali 63 omwe anatsukidwa mabala ku zipatala zazikulu za maboma, 47 (75%) anatsukidwa mabala pobaya kapena posabaya makhwala ochepesa ululu .kutsatira kuchepa machitidwe azinthu pa masabata asanu ndi chimodzi potsatira kuvulala kwa atenga mbali omwe anali ndi muyeso wa kuvulala zilonda zingonozingono ndi zapakatikati (Gustilo 1,11) ntchito ndi moyo wa bwino sunabwelele chimake ndi kuvulala kwa zilonda zazing'ono zing'ono (Gustillo I/II) ((posterior mean SMFA at 1 year: 10·5, 95% HDI: 9·5-11·6; QALYs: 0·73, 95% HDI: 0·66-0·80) kapena kuvulala kwa zilonda zikulu zikulu (Gustilo grade III) SMFA: 14·9, 95% HDI: 13·4-16·6; QALYs: 0·67, 95% HDI: 0·59-0·75). muyezo onse obvulala, kuyika kwa zitsulo za nkati kunasitha magwilidwe ntchito komaso miyoyo ya anthu omwe anatenga nawo mbali patadusa chaka chimodzi kuchokera tsiku lobvulala. Kuchedwa kuyika zitsulo zolumikizitsila mwendo za panja patadusa masiku asanu kunali ndi kuthekela koyambisa kuonopngeka kwa mabala kuyelekeza ndi kusamala mabala mwachangu nkati kati mwa masiku awiri (kutsithidwa chiwelengero; 5·1, 95% CI: 1·8-16·1, P=0·02

Kutathauzira
akuluakulu omwe anathyoka mwendo fupa kutulukira M' Malawi ali ndi mabvuto ogwila ntchito komaso mioyo wosachita bwino patatha chaka kutsatila pomwe anavulala. Zipatala zikulu zikulu zosamalira anthu ovulala kumbali ya mafupa kumbali ya anthu ovulala mafupa (centralised orthopaedic surgical management), kuphatikizilapo kuyikilatu nsanga zitsulo zankati mwamafupa komaso zitsulo zapanja zitha kusitha zotsatira za anthu omwe anavulala kwambiri
