# Supplementary material for: Functional outcomes and quality of life at 1-year follow-up after an open tibia fracture in Malawi: a multicentre, prospective cohort study
Source: Lancet Glob Health. 2023 Sep 1;11(10):e1609–18. doi: 10.1016/S2214-109X(23)00346-7 (PMC10509037; doi:10.1016/S2214-109X(23)00346-7)
Supplement: Supplementary appendix 2 [file mmc2.pdf]

THE LANCET
Global Health

Supplementary appendix 2

This appendix formed part of the original submission and has been peer reviewed. We post it as supplied by the authors.

Supplement to: Schade AT, Sabawo M, Nyamulani N, et al. Functional outcomes and quality of life at 1-year follow-up after an open tibia fracture in Malawi: a multicentre, prospective cohort study. *Lancet Glob Health* 2023; published online Sept 1. https://doi.org/10.1016/S2214-109X(23)00346-7.

Appendix

Supplemental material to manuscript:

Functional outcomes and quality of life at one year after an open tibia fracture in Malawi: a multicentre prospective cohort study

Schade AT MPH[1,2,3], Sabawo M BSc[4], Nohakhelha Nyamulani FCS ECSA (Tr & Ortho)[3], Mpanga Chiku MMed (Tr & Ortho)[3,4], Ngoie LB PhD[5], Prof Metcalfe A PhD[6], Prof Lalloo DG MD[2], Prof Madan JJ PhD[6], Harrison WJ FRCS (Tr & Ortho)[7,8], Prof MacPherson P PhD[1,2, 9]

1. Malawi-Liverpool-Wellcome Trust, Blantyre, Malawi
2. Liverpool School of Tropical Medicine, Liverpool, United Kingdom
3. Queen Elizabeth Central Hospital, Blantyre, Malawi
4. Kamuzu University of Health Sciences, Blantyre, Malawi
5. Kamuzu Central Hospital, Lilongwe, Malawi
6. University of Warwick Medical school, Coventry, United Kingdom
7. Countess of Chester NHS Foundation Trust, Chester, United Kingdom
8. AO Alliance, Davos, Switzerland
9. University of Glasgow, Glasgow, United Kingdom

Corresponding author: Alexander Thomas Schade (MPH), alexander.schade@lstmed.ac.uk

Appendix 1
1. Full search terms for the systematic review 2
2. Supplementary information to methods 6
3. Supplemental information to results..9
4. Outcomes 9
5. References 12

1. Full search terms for the systematic review

1. exp TIBIA/ or tibia*.mp. (91808)
2. exp Leg/ or lower limb*.mp. or exp Lower Extremity/ (177978)
3. 1 or 2
4. fracture*.mp. (247550)
5. exp Fractures, Open/ or compound fracture*.mp. or exp Fractures, Bone/ or exp Tibial Fractures/ (170535)
6. injur*.mp. (1005413)
7. trauma*.mp. (335417)
8. 4 or 5 or 6 or 7
9. exp Developing Countries/ or developing count*.mp. (107697)
10. low income country*.mp. (514)
11. Afghanistan.mp. or AFGHANISTAN/ (5386)
12. Albania.mp. or ALBANIA/ (1048)
13. Algeria.mp. or ALGERIA/ (3302)
14. Angola.mp. or ANGOLA/ (1268)
15. (Antigua and Barbuda).mp. (126)
16. Argentina.mp. or ARGENTINA/ (18039)
17. Armenia.mp. or ARMENIA/ (1575)
18. Azerbaijan.mp. or AZERBAIJAN/ (1509)
19. Bangladesh.mp. or BANGLADESH/ (11387)
20. Belarus.mp. or "Republic of Belarus"/ (2455)
21. Belize.mp. or BELIZE/ (757)
22. Benin.mp. or BENIN/ (3086)
23. Bhutan.mp. or BHUTAN/ (526)
24. Bolivia.mp. or BOLIVIA/ (3298)
25. (Bosnia and Herzegovina).mp. (2220)
26. Botswana.mp. or BOTSWANA/ (1966)
27. Brazil.mp. or BRAZIL/ (93963)
28. Burkina Faso.mp. or Burkina Faso/ (3556)
29. Burundi.mp. or BURUNDI/ (814)
30. Cabo Verde.mp. or Cabo Verde/ (204)
31. Cambodia.mp. or CAMBODIA/ (3712)
32. Cameroon.mp. or CAMEROON/ (6083)
33. Central African Republic.mp. or Central African Republic/ (1021)
34. Chad.mp. or CHAD/ (1063)
35. China.mp. or CHINA/ (172107)
36. Colombia.mp. or COLOMBIA/ (11164)
37. Comoros.mp. or COMOROS/ (397)
38. "Democratic Republic of the Congo"/ or Democratic Republic of Congo.mp. (4015)
39. Congo.mp. or CONGO/ (12054)
40. Cook Islands.mp. (170)
41. Costa Rica.mp. or Costa Rica/ (4499)
42. Cote d'Ivoire.mp. or Cote d'Ivoire/ (3348)
43. ivory coast.mp. or Cote d'Ivoire/ (3353)
44. Cuba.mp. or CUBA/ (5947)
45. Djibouti.mp. or DJIBOUTI/ (346)
46. Dominica.mp. or DOMINICA/ (371)
47. Dominican Republic.mp. or Dominican Republic/ (1939)
48. Ecuador.mp. or ECUADOR/ (4148)

49. EGYPT/ or Egypt.mp. (16883)
50. El Salvador.mp. or El Salvador/ (1228)
51. Equatorial Guinea.mp. or Equatorial Guinea/ (385)
52. Eritrea.mp. or ERITREA/ or Ethiopia/ (10396)
53. Ethiopia.mp. or ETHIOPIA/ (11340)
54. Fiji.mp. or FIJI/ (1385)
55. Gabon.mp. or GABON/ (1813)
56. Gambia.mp. or GAMBIA/ (2831)
57. "GEORGIA (REPUBLIC)"/ or GEORGIA/ or Georgia.mp. (14934)
58. Ghana.mp. or GHANA/ (8043)
59. Grenada.mp. or GRENADA/ (273)
60. Guatemala.mp. or GUATEMALA/ (3571)
61. GUINEA/ or EQUATORIAL GUINEA/ or GUINEA-BISSAU/ (1995)
62. Guyana.mp. or GUYANA/ (1018)
63. Haiti.mp. or HAITI/ (3425)
64. Honduras.mp. or HONDURAS/ (1582)
65. India.mp. or INDIA/ (109796)
66. Indonesia.mp. or INDONESIA/ (11760)
67. Iran.mp. or IRAN/ (24975)
68. IRAQ/ or Iraq.mp. (8131)
69. Jamaica.mp. or JAMAICA/ (4051)
70. Jordan.mp. or JORDAN/ (5059)
71. Kazakhstan.mp. or KAZAKHSTAN/ (2889)
72. Kenya.mp. or KENYA/ (17016)
73. Kiribati.mp. or Micronesia/ (1153)
74. Democratic People's Republic of Korea.mp. or "Democratic People's Republic of Korea"/ (225)
75. Kosovo.mp. or Yugoslavia/ or KOSOVO/ (9349)
76. Kyrgyzstan.mp. or KYRGYZSTAN/ (1360)
77. Laos/ or Lao People's Democratic Republic.mp. (1739)
78. Lebanon.mp. or LEBANON/ (4518)
79. Lesotho.mp. or LESOTHO/ (566)
80. Liberia.mp. or LIBERIA/ (1498)
81. Libya.mp. or LIBYA/ (1325)
82. Former Yugoslav Republic of Macedonia.mp. or "Macedonia (Republic)"/ (512)
83. Madagascar.mp. or MADAGASCAR/ (4254)
84. Malawi.mp. or MALAWI/ (5539)
85. Malaysia.mp. or MALAYSIA/ (15984)
86. Maldives.mp. or Indian Ocean Islands/ (830)
87. Mali.mp. or MALI/ (3205)
88. Marshall Islands.mp. or Micronesia/ (1172)
89. Mauritania.mp. or MAURITANIA/ (580)
90. Mauritius.mp. or MAURITIUS/ (845)
91. MEXICO/ or Mexico.mp. (48744)
92. Moldova.mp. or MOLDOVA/ (868)
93. Mongolia.mp. or MONGOLIA/ (3321)
94. Montenegro.mp. or MONTENEGRO/ (665)
95. Montserrat.mp. or West Indies/ (3443)
96. Morocco.mp. or MOROCCO/ (6253)
97. Mozambique.mp. or MOZAMBIQUE/ (2876)
98. Myanmar.mp. or MYANMAR/ (2786)

99. Namibia.mp. or NAMIBIA/ (1321)
100. Nauru.mp. or Micronesia/ (1145)
101. Nepal.mp. or NEPAL/ (8036)
102. Nicaragua.mp. or NICARAGUA/ (1787)
103. NIGER/ or Niger.mp. (10903)
104. Nigeria.mp. or NIGERIA/ (28539)
105. Niue.mp. or Polynesia/ (1791)
106. Pakistan.mp. or PAKISTAN/ (17419)
107. Palau.mp. or PALAU/ (339)
108. PANAMA/ or Panama.mp. (3731)
109. Papua New Guinea.mp. or Papua New Guinea/ (4695)
110. Paraguay.mp. or PARAGUAY/ (1311)
111. Peru.mp. or PERU/ (10084)
112. Philippines.mp. or PHILIPPINES/ (9903)
113. Rwanda.mp. or RWANDA/ (2554)
114. Saint Helena.mp. or Atlantic Islands/ (733)
115. Samoa.mp. or SAMOA/ (940)
116. (Sao Tome and Principe).mp. (116)
117. Senegal.mp. or SENEGAL/ (6669)
118. Serbia.mp. or SERBIA/ (4174)
119. Sierra Leone.mp. or Sierra Leone/ (1798)
120. Solomon Islands.mp. or Melanesia/ (1208)
121. Somalia.mp. or SOMALIA/ (1854)
122. South Africa.mp. or South Africa/ (43195)
123. South Sudan.mp. or South Sudan/ (327)
124. Sri Lanka.mp. or Sri Lanka/ (6546)
125. Saint Lucia.mp. or Saint Lucia/ (108)
126. (Saint Vincent and the Grenadines).mp. (56)
127. SUDAN/ or sudan.mp. (7546)
128. Suriname.mp. or SURINAME/ (1016)
129. Swaziland.mp. or SWAZILAND/ (712)
130. Syria/ or Syrian Arab Republic.mp. (1456)
131. Tajikistan.mp. or TAJIKISTAN/ (847)
132. Tanzania.mp. or TANZANIA/ (11676)
133. Thailand.mp. or THAILAND/ (29960)
134. Timor-Leste/ or Timor-Leste.mp. (269)
135. Togo.mp. or TOGO/ (1370)
136. Tokelau.mp. or Polynesia/ (1805)
137. Tonga.mp. or TONGA/ (403)
138. Tunisia.mp. or TUNISIA/ (8547)
139. TURKEY/ or Turkey.mp. (43289)
140. Turkmenistan.mp. or TURKMENISTAN/ (682)
141. Tuvalu.mp. or Micronesia/ (1117)
142. Uganda.mp. or UGANDA/ (12419)
143. Ukraine.mp. or UKRAINE/ (16266)
144. vanuatu.mp. or VANUATU/ (555)
145. venezuela.mp. or VENEZUELA/ (6193)
146. VIETNAM/ or vietnam.mp. (14498)
147. (wallis and futuna).mp. (30)
148. west bank.mp. (482)
149. Gaza.mp. (910)

150.yemen.mp. or YEMEN/ (1682)
151.zambia.mp. or ZAMBIA/ (4876)
152.zimbabwe.mp. or ZIMBABWE/ (6414)
153. OR/9-152
154. 3 and 8 and 153

2. Supplementary information to methods

Context

In Malawi, fracture care is delivered at 25 government rural district hospitals (one per district) and 4 urban government central hospitals (one per region). Surgical capacity in Malawi has been contextualised elsewhere (2). In short, each district hospital has between 1-3 orthopaedic clinical officers, 1-3 anaesthetic clinical officers, one major theatre, and one minor theatre. Only two out of the four central hospitals (Kamuzu Central Hospital, KCH and Queen Elizabeth Central Hospital, QECH) have permanent orthopaedic surgeons. The hospitals below are the only governmental hospitals in the area, there are private and faith-based hospitals, but very few people have healthcare insurance or can afford private healthcare (3).

**Recruitment site**	**Characteristics**
Kamuzu Central Hospital (KCH)	Central government hospital, Lilongwe (capital)<br>800 beds, catchment population ~4 million<br>~60 open tibia admissions per year (2018)<br>3 orthopaedic surgeons, 5 trainee orthopaedic surgeons<br>8 orthopaedic clinical officers<br>3 orthopaedic physiotherapists<br>1 anaesthetic trainees (orthopaedic specific)
Queen Elizabeth Central Hospital (QECH)	Central government hospital with NGO input, Blantyre<br>1000 beds, catchment population ~1 million<br>~74 open tibia admissions per year (2018)<br>3 orthopaedic surgeons, 4 trainee orthopaedic surgeons<br>5 orthopaedic clinical officers<br>3 orthopaedic physiotherapists

	2 anaesthetic trainees (orthopaedic specific)
Ntcheu District Hospital (ND)	District government hospital, Ntcheu<br><br>255 beds, catchment population ~529,000<br><br>Estimated ~15 open tibia admissions per year (2018)<br><br>3 orthopaedic clinical officers<br><br>6 physiotherapists (shared)<br><br>2 anaesthetists (shared)
Machinga District Hospital (MD)	District government hospital, Liwonde<br><br>140 beds, catchment population ~300,000<br><br>Estimated ~15 open tibia admissions per year (2018)<br><br>2 orthopaedic clinical officers<br><br>4 physiotherapists (shared)<br><br>3 anaesthetic clinical officers (shared)
Balaka District Hospital (BD)	District government hospital, Balaka<br><br>150 beds, catchment population ~500,000<br><br>Estimated ~15 open tibia admissions per year (2018)<br><br>3 orthopaedic clinical officers<br><br>4 physiotherapists (shared)<br><br>1 anaesthetic clinical officers (shared)
Dedza District Hospital (DD)	District government hospital, Dedza<br><br>150 beds, catchment population ~500,000<br><br>Estimated ~15 open tibia admissions per year (2018)<br><br>3 orthopaedic clinical officers<br><br>4 physiotherapists (shared)<br><br>3 anaesthetic clinical officers (shared)

Supplemental Table 1: characteristics of study sites

Procedures, outcomes, and follow-up

If clinical photographs were missing, documentation of wounds was reviewed by AS. Any uncertainties, such as borderline proximal or distal fractures, were reviewed by WJH. Where Gustilo grade was missing, AS undertook review of radiographs and clinical photographs and/or wound descriptions to classify fractures. We didn't include antibiotic use and treatment decisions in infection classification as there is substantial empirical intervention not guided by microbiological investigation in Malawi. The loss to follow-up at 6 weeks was due to the fact that early in the study, some participants were not successfully traced. However, as soon as we identified this issue, immediate action was taken to rectify the situation. The necessary measures were implemented to trace the participants, obtain their updated contact details, and ensure proper follow-up for the remaining study period, meaning that they attended later assessments.

Supplemental information to statistical analysis

Models were fit using the R `brms` package as an interface to CmdStanR in R (4). We rescaled SMFA scores to range between 0 and 1, and modelled outcome variables using zero-one-inflated beta distributions to capture excess extreme values (i.e. before injury, nearly all participants reported SMFA scores of 0 and EQ-5D-3L scores of 1). Priors were weakly informative. We included participant-level random intercepts, and adjusted for age, and days to first surgical intervention *apriori*. 2,000 post warmup posterior samples were drawn and summarised by their mean and 95% highest density interval. Models were checked by inspecting chain mixing, Gelman-Rubin statistics, and posterior predictive plots. As participants had multiple observations over the follow-up period, we included a random intercept term for each participant to reflect the temporal autocorrelation in their function and quality of life outcomes. As repeated measurements from participant are likely to be more similar to each other than measurements from different participants, this correlation was explicitly modelled (e.g. see Detry and Ma, JAMA 2016: "Analysing repeated measures using mixed models". doi:10.1001/jama.2015.19394s. for an overview of this statistical approach(5)).

3. Supplemental information to results

The eight participants that left hospital before recruitment did so due to refusal to be transferred to the COVID-19 isolation ward.

Mechanisms of injuries differed between the tertiary and district hospitals, with more assaults presenting to tertiary hospitals compared to district hospitals (18% vs 6%) and more blunt force and sport injuries in district hospitals. There were more Gustilo grade III injuries in tertiary hospitals (112/218, 51%) compared to district hospitals (15/63, 24%, p<0·01). Overall, 26% (76/287) of fractures required Gustilo classification by review of clinical photographs and radiographs. Most, 185/186 (99·5%), operative fixations were performed in the tertiary hospitals.

4. Outcomes

Model trace plots showed good convergence and Gelman-Rubin statistics for all parameters were <1·01. For participants with Gustilo grade I/II factures, age at the time of injury had a substantial impact on recovery of function and quality of life over the subsequent year (Supplemental Figure 1). For example, participants aged 18 years (week 52 SMFA: 7.2, 95% HDI: 3·8 to 10·9; week 52 EQ-5D-3L: 0·86, 95% HDI: 0·81 to 0·90) had better recovery than participants aged 65 years (week 52 SMFA: 13·2, 95% HDI: 7·1 to 20·4; week 52 EQ-5D-3L: 0·78, 95% HDI: 0·71 to 0·85; posterior mean difference in SMFA: -5·97, 95% HDI: -10·5 to -2·0; posterior mean difference in EQ-5D-3L: 0·08, 95% HDI: 0·01 to 0·14), with a clear gradient across age groups. However, for the more severe Gustilo grade III injuries, younger age did not appear to have a positive effect on recovery (posterior mean difference in SMFA at one year: -4·01, 95% HDI: -9.9·0 to 1·9; posterior mean difference in EQ-5D-3L: 0·02, 95% HDI: -0·04 to 0·10). Similar trends were seen for the effect of time to initial surgical management, with worse function and quality of life with longer treatment delays (Supplemental Figure 2).

Supplemental Figure 1: Effect of age at time of open tibia fracture on recovery of function and quality of life over one year

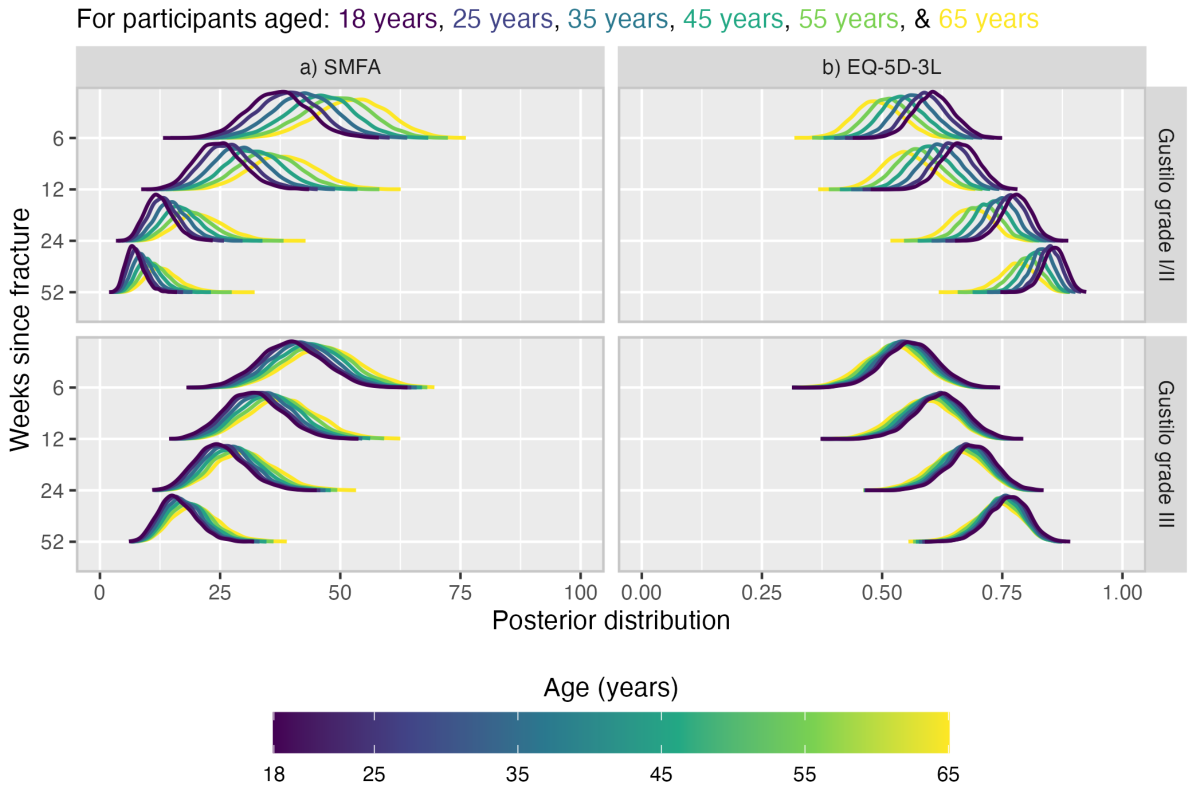

Modelled with days to initial surgical intervention held constant at its median

Supplemental Figure 2: Effect of time to initial surgical intervention on recovery of function and quality of life over one year

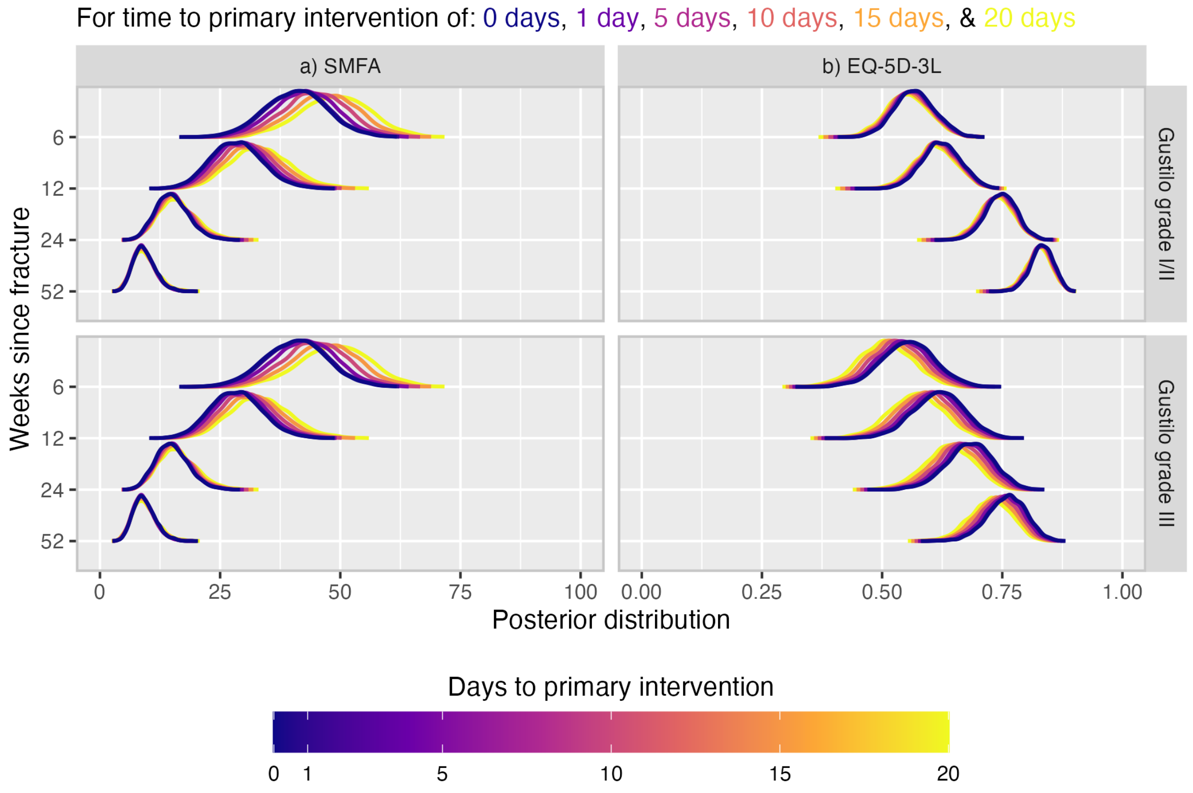

Modelled with age held constant at its mean, and participant-level random effects

5. References

1. Schade AT, Hind J, Khatri C, Metcalfe AJ, Harrison WJ. Systematic review of patient reported outcomes from open tibia fractures in low and middle income countries. Injury. 2020;51(2):142-6.
2. Henry JA, Frenkel E, Borgstein E, Mkandawire N, Goddia C. Surgical and anaesthetic capacity of hospitals in Malawi: key insights. Health Policy and Planning. 2014;30(8):985-94.
3. Wingston Felix Na, Farai C, Takondwa M, Agnes Jack B, Joseph M-B. Uptake of health insurance in Malawi in 2019-2020: Evidence from the Multiple Indicator Cluster Survey. medRxiv. 2022:2022.08.18.22278931.
4. Bürkner P-C. Brms: An R package for Bayesian multilevel models using Stan. J Stat Softw. 2017;80(1).
5. Detry MA, Ma Y. Analyzing Repeated Measurements Using Mixed Models. Jama. 2016;315(4):407-8.
