# Supplementary material for: Functional outcomes and quality of life at 1-year follow-up after an open tibia fracture in Malawi: a multicentre, prospective cohort study
Source: Lancet Glob Health. 2023 Sep 1;11(10):e1609–18. doi: 10.1016/S2214-109X(23)00346-7 (PMC10509037; doi:10.1016/S2214-109X(23)00346-7)
Supplement: Supplementary appendix 3 [file mmc3.pdf]

THE LANCET
Global Health

Supplementary appendix 3

This Equitable Partnership Declaration (EPD) was submitted by the authors, and we reproduce it as supplied. It has not been peer reviewed. The Lancet's editorial processes have not been applied to the EPD.

Supplement to: Schade AT, Sabawo M, Nyamulani N, et al. Functional outcomes and quality of life at 1-year follow-up after an open tibia fracture in Malawi: a multicentre, prospective cohort study. *Lancet Glob Health* 2023; published online Sept 1. https://doi.org/10.1016/S2214-109X(23)00346-7.

Equitable Partnership Declaration

Researcher considerations

1. Please detail the involvement that researchers who are based in the region(s) of study had during a) study design; b) clinical study processes, such as processing blood samples, prescribing medication, or patient recruitment; c) data interpretation; and d) manuscript preparation, commenting on all aspects. If they were not involved in any of these aspects, please explain why.

   This question is intended for international partnerships; if all your authors are based in the area of study, this question is not applicable.

   This should include a thorough description of their leadership role(s) in the study. Are local researchers named in the author list or the acknowledgements, or are they not mentioned at all (and, if not, why)? Please also describe the involvement of early career researchers based in the location of the study. Some of this information might be repeated from the Contributors section in the manuscript. Note: we adhere to [ICMJE authorship criteria]() when deciding who should be named on a paper.

a) Study design:

MS is a Malawian orthopaedic clinical officer
NN and MC are Malawian orthopaedic surgeons
NB is a Congolese orthopaedic surgeon who has been working in Malawi for >25 years

MS, NN, MC and NB were all involved in the design of the study. Furthermore, they organized multiple stakeholder meetings within Malawi to engage local professionals such as other Malawian orthopaedic surgeons, anaesthetists, clinical officers, nurses, and patients. These meetings were aimed to gather feedback and incorporate the Malawian orthopaedic community's perspective in the design.

The researchers also sought feedback on the study design from each district hospital's health officer, emphasizing the importance of local healthcare facilities in shaping the study design. This collaborative approach allowed the researchers to gain insights into the challenges and unique characteristics of the region. To ensure wider dissemination of the study protocol and receive input from a broader range of stakeholders, MB shared the protocol with the Malawi Orthopaedic Association executive committee to ensure perspectives from and collaboration with clinical officers from other district hospital sites.

Finally, in order to receive approval for the Malawian study ethics, we changed the study design based on feedback from the college of medicine ethics and research committee (COMREC) and letters of support from each the district hospital management teams (usually 10 people). This process highlights the commitment to addressing ethical considerations and aligning the study design with the requirements and preferences of the Malawian context.

b) Clinical study processes:

MS, NN, CM and NB were all involved in the acquisition of the data. Their expertise and involvement ensured that the clinical study processes were carried out with proper medical guidance and adherence to established protocols. Specifically, in terms of patient recruitment, the surgeons played

a crucial role in the selection process. Eligible cases were discussed amongst the surgeons to ensure that the criteria for recruitment were met. MS provided valuable feedback on eligible participants from other clinical officers working in the district hospitals. This collaboration and sharing of knowledge helped to enhance the overall quality of the clinical study processes, ensuring a comprehensive and informed approach to patient care and research.
**c) Data interpretation:**<br><br>Preliminary data was presented to the orthopaedic department approximately every 4 months to facilitate discussions and gather valuable insights. MB, NN, CM, and LB carefully examined the preliminary data and provided their suggestions on variables to analyse and include in the manuscript. Their expertise and knowledge in the field of orthopaedics allowed them to offer valuable perspectives on which variables should be considered for analysis. By collectively reviewing the data, discussing the findings, and providing input, the researchers ensured a comprehensive and rigorous approach to the interpretation of the data. The collaborative nature of this process, involving researchers based in the region, helped to enrich the data interpretation phase, and ensure that the analysis captured the most relevant and significant aspects of the study.
**d) Manuscript preparation:**<br><br>AS and PM took the lead in drafting the initial version of the manuscript. However, they received significant support from MB, NN, CM, and LB in specific sections, such as the introduction and discussion. This collaborative approach allowed for a well-rounded representation of different perspectives and expertise, strengthening the overall quality of the manuscript.

2. Were the data used in your study collected by authors named on the paper, or have they been extracted from a source such as a national survey? ie, is this a secondary analysis of data that were not collected by the authors of this paper. If the authors of this paper were not involved in data collection, how were data interpreted with sufficient contextual knowledge?

   The Lancet Global Health *believe contextual understanding is crucial for informed data analysis and interpretation.*

MS, NN, CM and LB all played a vital role in data analysis for this prospective cohort study. To inform data analysis, preliminary findings were presented every 4 months at the orthopaedic departmental meetings at the tertiary hospitals.

3. How was funding used to remunerate and enhance the skills of researchers and institutions based in the area(s) of study? And how was funding used to improve research infrastructure in the area of study?

   Potentially effective investments into long-term skills and opportunities within institutions could include training or mentorship in analytical techniques and manuscript writing, opportunities to lead all or specific aspects of the study, financial remuneration rather than requiring volunteers, and other professional development and educational opportunities.

Improvements to research infrastructure could be funding of extended trial designs (such as platform trials) and use of master protocols to enable these designs, establishment of long-term contracts for research staff, building research facilities, and local control of funding allocation.

**Skills:**<br><br>Funding plays a crucial role in supporting researchers and institutions in their pursuit of knowledge and innovation. The Wellcome Trust has been instrumental in using its funding to remunerate and enhance the skills of researchers by funding research training courses for Malawian clinical and research staff (including study team members) such as Good Clinical Practice or introduction to Statistics at the Malawi-Liverpool-Wellcome Trust in Blantyre, Malawi.<br><br>Furthermore, Wellcome Trust's funding, through a collaboration with the AO Alliance and AO Trauma, provided support to tuition fees for MS master's projects in the area of study (Master of Science in Global Health Implementation). This financial support encourages the development of specialized skills and expertise amongst local researchers. This study also allocated funds for a capacity-building event (Malawi Orthopaedic Association Annual General Meeting) among the orthopaedic clinical officers once a year. This event aimed to improve the capabilities and skills of researchers and institutions by providing them with training, workshops, and resources to enhance their research capabilities.
**Research infrastructure:**<br><br>As part of the study's infrastructure development, a disused space was renovated by the study team to establish the first orthopaedic research offices. This renovation involved utilizing study funds to create a suitable working environment for research activities. The process involved collaboration between MLW and the hospital management team through a rental agreement. By repurposing an unused space, the study team was able to optimize existing resources and establish a dedicated research facility that could contribute to future research endeavours and benefit the orthopaedic department.

4. How did you safeguard the researchers who implemented the study?

Please describe how you guaranteed safe working conditions for study staff, including provision of appropriate personal protective equipment, protection from violence, and prevention of overworking.

To guarantee safe working conditions for study staff, the Malawi-Liverpool-Wellcome Trust complies with Malawian labour laws and regulations. These laws outline the rights and protections afforded to employees, including provisions related to working hours, rest periods, and occupational safety. By adhering to these laws, MLW ensures that study staff are treated fairly and their working conditions meet the required standards. These rights and protections are also monitored internally and externally by different committees.<br><br>Furthermore, MLW has implemented strict policies and protocols to protect the study staff, particularly in the context of COVID-19. As the global pandemic posed significant risks, MLW prioritized the safety of its staff by providing appropriate personal protective equipment (PPE). This

included masks, gloves, face shields, and all necessary equipment to minimize the transmission of the virus and safeguard the well-being of the study staff. There is also a COVID-19 committee that reviews and assists in responding to COVID-19 waves.

Benefits to the communities and regions of study

5. How does the study address the research and policy priorities of its location?

How were the local priorities determined and then used to inform the research question? Who decided which priorities to take forward? Which elements of the study address those priorities?

As part of the country initiative for Malawi, AO Alliance convened a needs assessment with local Malawian Surgeons, nurses and clinical officers in Liwonde, Malawi in 2014 and then a second needs assessment in Blantyre, Malawi in 2019. This aimed to understand the challenges, gaps, and requirements in the management of musculoskeletal injuries in Malawi. This assessment directly addressed the research and policy priorities of the location by identifying the specific areas where improvements are needed. The findings of the needs assessment can inform research and policy development, allowing for targeted interventions to address the identified gaps and enhance orthopaedic care in the country.

The preliminary qualitative research conducted at Queen Elizabeth Central Hospital (QECH) provided valuable insights into the experiences, perspectives, and challenges faced by surgeons and healthcare providers in managing open fractures in Malawi. This work directly addressed the research and policy priorities of the location by capturing the local context and factors influencing the management of open fractures. The findings from the qualitative research informed the data collection forms and methodology of the prospective cohort study.

The decision by the orthopaedic clinical officers to choose open fracture management as the topic for their organization's national meeting in 2022 demonstrates that it is a priority area in Malawi. The consensus meeting provides a platform for stakeholders to come together, share their knowledge, discuss challenges, and collaborate on finding solutions to improve the management of open fractures. This meeting directly addresses the research and policy priorities of the location by focusing on a specific area of concern identified by the healthcare professionals themselves. It facilitates the exchange of ideas, best practices, and experiences, which can inform policy recommendations and implementation strategies to enhance open fracture management in Malawi.

6. How will research products be shared in the community of study?

For instance, will you be providing written or oral layperson summaries for non-academic information sharing? Will study data be made available to institutions in the region(s) of study? The Lancet Global Health *encourages authors to translate the summary (abstract) into relevant languages after paper editing; do you intend to translate your summary?*

The study team collaborated with different media outlets including radio, television and newspapers to create a road traffic awareness week to share data with the general public. This partnership allowed for the dissemination of study findings through a platform that reaches a wide audience. By leveraging the media outlet's reach, the research team was able to share important information related to road traffic and raise public awareness about the study's findings. This approach ensures that research findings are communicated beyond academic circles and reach a broader population.

See:
https://malawiorthopaedic.mw/
https://twitter.com/Times360Malawi/status/1437360018584637440
https://www.youtube.com/watch?v=yjFbXkR7m2s&ab_channel=MalawiOrthopaedicAssociation

The study team also presented a short presentation and infographics of the research data to all the clinical officers at district hospitals. The information was displayed within the hospitals and presented during morning handovers, ensuring that the study findings are accessible to healthcare professionals working directly with patients. By sharing the data in a visual format and integrating it into their daily routines, the study aims to enhance understanding and application of the research findings in the clinical setting.

The study has also made efforts to share data with all the orthopaedic professionals involved in the research. This may include sharing the study findings through stakeholder presentations, workshops, or postgraduate meetings. By directly engaging with orthopaedic professionals, the study facilitates knowledge dissemination and encourages the uptake of research outcomes in their practice. This helps to bridge the gap between research and clinical practice, leading to improved patient care and outcomes.

7. How were individuals, communities, and environments protected from harm?

a) *How did you ensure that sensitive patient data was handled safely and respectfully? Was there any potential for stigma or discrimination against participants arising from any of the procedures or outcomes of the study?*

Since it was an observational study that primarily involved patient-reported outcomes, the potential for stigma arising from the study procedures or outcomes was minimal. However, the study team still implemented measures to handle sensitive patient data responsibly.

To ensure the safe handling of patient data, strict data security and confidentiality protocols were followed. This included maintaining encrypted and password-protected databases, restricting access to authorized personnel only, and adhering to relevant data protection regulations and guidelines. The study team took precautions to anonymize patient data during analysis and reporting, using unique identifiers instead of personally identifiable information. By employing these measures, the study aimed to protect the privacy and confidentiality of the participants' sensitive information.

Regarding potential stigma or discrimination, the study team was aware of the sensitive nature of open tibia fractures and the potential impact on patients' well-being and societal perceptions. In prior qualitative work, themes related to masculinity and stigma following an open tibia fracture, particularly in cases where amputation was involved, were explored. This qualitative research

aimed to gain insights into the experiences and perspectives of individuals affected by these fractures, addressing potential stigma and discrimination related to the outcomes. The findings from the qualitative work allowed the study team to better understand the social and psychological implications of open tibia fractures and identify potential areas where stigma or discrimination could occur. By addressing these aspects, the study aimed to raise awareness, mitigate potential stigma or discrimination, and contribute to a more compassionate and patient-centred approach to care

b) *Might any of the tests be experienced as invasive or culturally insensitive?*

These were patient-reported outcomes, where privacy was always emphasised to keep patients comfortable.

c) *How did you determine that work was sensitive to traditions, restrictions, and considerations of all cultural and religious groups in the study population?*

During the study design phase, the research team actively collaborated with the Malawi-Liverpool-Wellcome Trust Media and Communication department to obtain valuable insights and guidance on cultural and religious sensitivities that needed to be considered. This collaboration allowed the researchers to gain a deeper understanding of the cultural nuances and specific considerations of the study population. In the dissemination process, the Media and Communication department provided guidance on how the research findings should be communicated to the public in a culturally appropriate and sensitive manner. They helped ensure that the messaging was respectful and aligned with the traditions, restrictions, and considerations of all cultural and religious groups involved. This included guidance on using appropriate language, visuals, and channels of communication to effectively reach and engage different cultural and religious groups.

The involvement of the Malawi-Liverpool-Wellcome Trust ethics department also played a crucial role in ensuring that the study work respected and considered the ethical implications of working with diverse cultural and religious groups. The ethics department likely provided oversight and guidance on how to navigate sensitive issues and ensure that the study design and implementation adhered to ethical principles.

d) *Were biowaste and radioactive waste disposed of in accordance with local laws?*

Not applicable.

e) *Were any structures built that would have impacted members of the community or the environment (such as handwashing facilities in a public space)? If so, how did you ensure that you had appropriate community buy-in?*

In this study, no structures were built that would have impacted members of the community or the environment. The research activities were primarily conducted within the hospital setting, focusing on patient-reported outcomes and clinical management.

f) *How might the study have impacted existing health-care resources (such as staff workloads, use of equipment that is typically employed elsewhere, or reallocation of public funds)?*

To ensure the smooth implementation of the study and manage the increased workload, extra non-clinical staff were employed specifically for data collection. These additional staff members helped alleviate the burden on existing healthcare personnel, allowing them to focus on their clinical responsibilities while ensuring the study's data collection activities were carried out effectively.

All equipment used for the study, including personal protective equipment (PPE), was purchased using the study budget. This approach ensured that the study had dedicated resources for essential equipment and reduced the need to rely on equipment from a low-resource healthcare system. Any surplus materials or equipment that remained after the study's completion were donated to the orthopaedic department, further contributing to the enhancement of healthcare resources within the department.

8. Finally, please provide the title (eg, Dr/Prof, Mr/Mrs/Ms/Mx), name, and email address of an author who can be contacted about this statement. This can be the corresponding author.

Name: Dr Alexander Thomas Schade
Email: alexander.schade@lstmed.ac.uk
